# Dynamic electrophysiological mechanism in patients with long-standing persistent atrial fibrillation

**DOI:** 10.3389/fcvm.2022.953622

**Published:** 2022-09-29

**Authors:** Emilio Osorio-Jaramillo, James L. Cox, Sarah Klenk, Alexandra Kaider, Philipp Angleitner, Paul Werner, Andreas Strassl, Markus Mach, Guenther Laufer, Marek P. Ehrlich, Niv Ad

**Affiliations:** ^1^Department of Cardiac Surgery, Medical University of Vienna, Vienna, Austria; ^2^Division of Cardiac Surgery, Bluhm Cardiovascular Institute, Feinberg School of Medicine, Northwestern University, Chicago, IL, United States; ^3^Division of Cardiology, Clinic Favoriten, Vienna, Austria; ^4^Department of Cardiac Surgery, Informatics and Intelligent Systems, Medical University of Vienna, Vienna, Austria; ^5^Division of Cardiovascular and Interventional Radiology, Medical University of Vienna, Vienna, Austria; ^6^Cardiothoracic Surgery, Adventist HealthCare White Oak Medical Center, Silver Spring, MD, United States; ^7^Division of Cardiac Surgery, Johns Hopkins University, Baltimore, MD, United States

**Keywords:** atrial fibrillation, electrophysiology, non-invasive electrocardiographic imaging, sequential mapping, localization of AF drivers

## Abstract

**Background:**

Improved understanding of the mechanisms that sustain persistent and long-standing persistent atrial fibrillation (LSpAF) is essential for providing better ablation solutions. The findings of traditional catheter-based electrophysiological studies can be impacted by the sedation required for these procedures. This is not required in non-invasive body-surface mapping (ECGI). ECGI allows for multiple mappings in the same patient at different times. This would expose potential electrophysiological changes over time, such as the location and stability of extra-pulmonary vein drivers and activation patterns in sustained AF.

**Materials and methods:**

In this electrophysiological study, 10 open-heart surgery candidates with LSpAF, without previous ablation procedures (6 male, median age 73 years), were mapped on two occasions with a median interval of 11 days (IQR: 8–19) between mappings. Bi-atrial epicardial activation sequences were acquired using ECGI (CardioInsight™, Minneapolis, MN, United States).

**Results:**

Bi-atrial electrophysiological abnormalities were documented in all 20 mappings. Interestingly, the anatomic location of focal and rotor activities changed between the mappings in all patients [100% showed changes, 95%CI (69.2–100%), *p* < 0.001]. Neither AF driver type nor their number varied significantly between the mappings in any patient (median total number of focal activities 8 (IQR: 1–16) versus 6 (IQR: 2–12), *p* = 0.68; median total number of rotor activities 48 (IQR: 44–67) versus 55 (IQR: 44–61), *p* = 0.30). However, individual zones showed a high number of quantitative changes (increase/decrease) of driver activity. Most changes of focal activity were found in the left atrial appendage, the region of the left lower pulmonary vein and the right atrial appendage. Most changes in rotor activity were found also at the left lower pulmonary vein region, the upper half of the right atrium and the right atrial appendage.

**Conclusion:**

This clinical study documented that driver location and activation patterns in patients with LSpAF changes constantly. Furthermore, bi-atrial pathophysiology was demonstrated, which underscores the importance of treating both atria in LSpAF and the significant role that arrhythmogenic drivers outside the pulmonary veins seem to have in maintaining this complex arrhythmia.

## Introduction

Improved understanding of the mechanisms that sustain persistent and long-standing persistent atrial fibrillation (LSpAF) is essential for providing better ablation solutions. Traditionally, electrophysiological studies are mostly catheter-based; their findings can therefore be impacted and suppressed by the sedation required for these procedures. The subsequent ablative procedure can result in scarring, which may, in turn, alter a later mapping. Non-invasive body-surface mapping (electrocardiographic imaging—ECGI) does not entail such alterations.

ECGI is a relatively novel mapping tool which allows for simultaneous recording of bi-atrial AF activation sequences under physiological conditions. ECGI requires wrapping the patient’s chest in a multi-electrode vest, but does not require sedation allowing for multiple mappings in the same patient at different times. ECGI has been used clinically to aid interventional ablation procedures, but it has so far been used only for single mappings (1, 2). Interestingly, Haïssaguerre and colleagues (1) have reported on a subset of 10 patients with repeated ECGI mapping after 6 h, which they say showed stable main AF driver regions. At the same time however, the group observed that rotor activity was meandering with the core variably traveling over an area of 7 ± 2 cm^2^. They conclude that reentrant driver locations must be described not as discrete sites, but more broadly as regions.

Other studies, manly basic-research, have previously shown that AF drivers have the ability to meander. Zlochiver et al. recorded meandering rotors as AF sources in isolated sheep hearts and in simulated 2D models of human atrial tissue (3). Another group by Roy et al. constructed left atrial models with patient-specific geometry and fibrosis distribution derived from late gadolinium enhanced magnetic resonance imaging of 6 AF patients. Their data suggests that the dynamic of re-entrant drivers is strongly influenced by the spatial distribution of fibrosis (4). Richard Schuessler and colleagues have elegantly shown during a short period of intraoperative epicardial mapping that the location of dominant frequency changed during the recording period in 48% of the patients. However, they assumed that due to the limited sampling, longer periods of recording would increase that percentage (5). Hansen et al. also acknowledged that temporal stability of AF driver patterns can be highly variable, which is why they used adenosine challenge in explanted human hearts of patients with a history of persistent atrial fibrillation to transiently stabilize reentrant drivers in order to unmask so-called arrhythmogenic hubs (6).

We hypothesized that ECGI would be able to expose potential electrophysiological changes over time, such as the location and stability of extra-pulmonary vein drivers and activation patterns in sustained AF. Sequential ECGI mapping might bear the potential to improve our understanding of the mechanisms that sustain persistent and long-standing persistent atrial fibrillation.

## Materials and methods

Approval for the study was obtained from the Ethics Review Board of the Medical University of Vienna. The study was conducted in accordance with the principles of the Declaration of Helsinki. All patients gave written informed consent prior to participation in the study.

**FIGURE 1:**
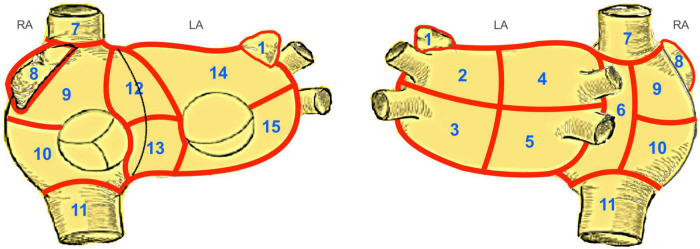
Modified Bordeaux Atrial Region Classification. The original Bordeaux atrial region classification (1) divides the CT-based atrial geometry into seven regions to provide distinct anatomical classification. We modified this classification keeping the same principles of bi-atrial regional subdivision into 15 zones guided by endocardial and epicardial ablation schemes. Zones 1–5, 14 and 15 correspond to the left atrium. Zones 12 and 13, the anterior interatrial groove, covers a region between both atria, belonging equally to both. The zones 6–11 correspond to the right atrium. LA, left atrium; RA, right atrium; 1, left atrial appendage; 2, region of left upper PV; 3, region of left lower PV; 4, region of right upper PV; 5, region of right lower PV; 6, posterior interatrial groove; 7, inflow of superior vena cava; 8, right atrial appendage; 9, upper region of right atrium; 10, lower region of the right atrium; 11, inflow of inferior vena cava; 12, upper half of anterior interatrial groove; 13, lower half of anterior interatrial groove; 14, upper anterior region of the left atrium; 15, lower anterior region of the left atrium.

Ten consecutive patients with LSpAF and no previous catheter ablation scheduled for cardiac surgery between February and December 2020 underwent extensive non-invasive electrocardiographic imaging (ECGI) on two separate dates prior to the surgical intervention. A minimum time interval of 7 days between the two electrophysiological studies was mandatory. No change of medication or other interventions were allowed, neither during mapping itself nor between the first and second mapping. Six patients were male, and the median age of the 10 patients was 73 years (IQR: 62–78). All patients had LSpAF (7, 8). The median duration of AF was 90 months (IQR: 41–195). The majority were candidates for concomitant mitral valve surgery and surgical ablation. Table 1 depicts the patient characteristics. The original Bordeaux atrial region classification (1) was modified keeping the same principles of bi-atrial regional subdivision into 15 zones guided by endocardial and epicardial ablation schemes (Figure 1).

**TABLE 1:** Patient characteristics.

Variables	All patients (*n* = 10)
Age, years, median (IQR)	73 (62–78)
Female sex	3 (30)
Weight, kg, median (IQR)	85 (65–97)
Height, cm, median (IQR)	172 (163–176)
Atrial fibrillation duration, months, median (IQR)	90 (41–195)
Long-standing persistent AF	10 (100)
Antiarrhythmic drugs	
Bisoprolol	5 (50)
Digitalis and Bisoprolol	2 (20)
Amiodarone	0 (0)
None	3 (30)
Concomitant cardiac disease	
MVR	1 (10)
MVR + TVR	5 (50)
MVR/MVS + TVR	1 (10)
AVR + AAA + CAD	1 (10)
AVS + CAD	1 (10)
AVS (prosthesis) + LVOTO	1 (10)
Previous cardiac surgery	2 (20)
Ejection fraction	
> 55%	9 (90)
45–55%	0 (0)
35–45%	1 (10)
NYHA-classification	
I	3 (30)
II	5 (50)
III	2 (20)
IV	0 (0)
Left atrial size, mm, median (IQR)	50 (48–58)
Right atrial size, mm, median (IQR)	60 (53–68)
Diabetes mellitus	2 (20)
COPD	2 (20)

Values are *n* (%) unless indicated otherwise.

AAA, aneurysm of the ascending aorta; AF, atrial fibrillation; AVR, aortic valve regurgitation; AVS, aortic valve stenosis; CAD, coronary artery disease; COPD, chronic obstructive pulmonary disease, IQR, interquartile range; LVOTO, left ventricular outflow tract obstruction; NYHA, New York Heart Association, TVR, tricuspid valve regurgitation.

The following power calculation was performed prior to commencement of the study and was based on the one group χ^2^-test: By including 10 patients, the null hypothesis of a 10% proportion of patients with changes of the location or quantity of focal and rotor activity can be rejected with a power of 90%, if the true underlying proportion is 50%, which was defined as the alternative hypothesis (null hypothesis: π0 = 10%; alternative hypothesis: π1 = 50%). The significance level was set at α = 0.05.

Descriptive statistical methods were applied to describe the study population and results. Absolute numbers (percentages) are reported for categorical variables and medians (interquartile ranges) in case of continuous variables.

Contingency tables were used to discern potential electrophysiological changes between both mapping time-points. Patients presenting with *driver activity* in the first mapping and later with *no activity* in the second mapping, or vice versa, in at least one of the zones would be considered patients with a change. The percentage of patients with a change is given together with the 95% confidence interval and the *p*-value resulting from the one group χ^2^-test, testing the null hypothesis of a 10% proportion.

For the quantitative analyses the median number of rotor and focal activities were calculated for each zone and overall. The quantitative mapping results were compared with the paired Wilcoxon signed-rank test. Two-sided *p*-values < 0.05 were defined as statistically significant. Statistical analyses were performed using SAS version 9.4 (SAS Institute Inc., Cary, NC, United States).

### Mapping technique

**FIGURE 2:**
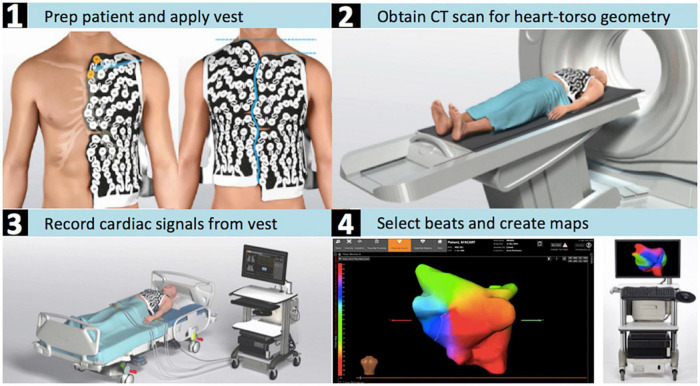
CardioInsight™ mapping system. Depicted are the steps required for the estimation of the electrical potentials on the atrial epicardial surface with the aid of non-invasive electrocardiographic imaging. **(1)** The patient is shaved and the ECGI electrode vest is put on the dry skin. **(2)** The patient’s torso and cardiac geometries are acquired through computed tomography while wearing the vest. **(3)** Body-surface potentials are recorded with an array of 252 body-surface electrodes. **(4)** 3D reconstruction of both atria is performed and the atrial surface potentials are reconstructed by the system’s algorithms.

Acquisition of electrical signals and subsequent computational methods for the reconstruction of non-invasive mappings was carried out using the CardioInsight™ non-invasive 3D-Mapping System (Medtronic Inc., Minneapolis, MN, United States) (9). In order to record body-surface potentials, a 252-electrode vest was applied to the patient’s torso (Figure 2). A minimum recording time of 10 s was used to allow for reliable processing and interpretation of the data. Recording was followed by a thoracic CT scan to obtain high-resolution images of the heart and the vest electrodes (3rd-Generation Dual-Source CT, SOMATOM Force, Siemens Healthineers, Forchheim, Germany). After the CT scan 3D epicardial bicameral atrial geometries can be reconstructed from segmental CT images in a semi-automated fashion on the ECGI mapping system. Segmentation was performed by manually marking the outer limits of the atria on the acquired cross-sectional images of the atrium on the system in all cases in order to increase accuracy of segmentation. The relative positions of body-surface electrodes were visualized on the torso geometry. The system reconstructs epicardial potentials, unipolar electrograms, potential activation and directional activation maps in every cardiac cycle using preconstructed mathematical reconstruction algorithms (10–12).

Phase map analysis was used for processing of the acquired signals to identify wave-front propagation *via* assessment of the whole morphology of each signal (13).

Baykaner et al. defined the term “AF driver” as electrically mappable mechanism that sustains, rather than initiates, fibrillatory conduction (14). In our and previous studies, (1, 2, 15) that have worked with the CardioInsight system, the term “AF driver” is used for both electrical mechanisms, that can be non-invasively mapped with this system. The system is able to detect two types of potential drivers of AF *via* phase analysis: focal activations and circulating wavefronts and categorizes them as focal and rotor activity. All detections have spatial/temporal constraints applied in the system. An activation wavefront will only be identified by the system and categorized as a potential “driver” if it performs a rotational movement and it needs to rotate around a stable core.

The exact definition of focal and rotor activity detection by the CardioInsight system is as follows:

• Focal activity is defined as an activation arising from a single stable point radiating outward.
• Rotor activity is defined as a wave rotating a minimum of 1.5 times around a spatially stable core. A rotor is only kept by the system if the core does not meander more than 2 cm in diameter and rotated at least 1.5 rounds (1).

A minimum T-R interval of more than 800 ms is necessary to perform appropriate mapping and reliable data recording in phase mapping (15–17). Sometimes pharmacological intervention (e.g., beta-blockade) is needed to slow down the heart rate, but this has not been the case in our patient cohort. Recordings were sent to a blinded core laboratory for processing (blinded concerning patients and the respective recording sequence). Processing included a thorough signal-quality check: Individual electrodes, showing aberrant signals, were manually excluded prior to calculation of the phase maps. Phase map analysis was followed by a manual review of the reconstructed unipolar electrograms to exclude noisy signals/artifacts. Every rotational activity was checked for plausibility during review and confirmed after sequential activation of the unipolar electrograms covering the local cycle length around a pivot point. Focal activity detections were reviewed to remove potential detections due to far-field projections.

## Results

All patients successfully underwent ECGI twice. The median time interval between the two mappings was 11 days (IQR: 8–19 days). A total of 142 and 144 phase windows were analyzed at the first and second mapping respectively, resulting in a total recording time of 147 s and 142 s, respectively. The median analyzed time per patient was 15 s in both mappings (Table 2).

**TABLE 2:** Overall number of drivers.

	All mappings (*n* = 20)	First mapping (*n* = 10)	Second mapping (*n* = 10)
Time analyzed, ms, median (IQR)	15,317 (14,334–15,601)	15,048 (14,420–15,707)	15,317 (14,238–15,943)
**Focal activity**			
Cumulative number	170 (100)	89 (52)	81 (48)
Median number (IQR; min, max)	7 (2–15; min: 0, max: 24)	8 (1–16; min: 0, max: 24)	6 (2–12; min: 0, max: 20)
Patients with focal activity	17 (85)	8 (80)	9 (90)
**Rotor activity**			
Cumulative number	1,005 (100)	509 (51)	496 (49)
Median number (IQR; min, max)	51 (44–62; min: 5, max: 70)	48 (44–67; min: 31, max: 70)	55 (44–61; min: 5, max: 66)
Patients with rotor activity	20 (100)	10 (100)	10 (100)

Values are *n* (%) unless indicated otherwise.

### Distribution of rotor and focal activity

In all subjects and all 20 mappings a bi-atrial arrhythmogenic pathology was identified. In total, 509 rotors and 89 focal triggers were identified and located during the first mapping, and 496 rotors and 81 focal triggers in the second mapping (Table 2).

Focal activity was detected in 17 of 20 mappings (85%). The majority of focal activities was detected in both the right and left atrial appendages (zones 1 and 8 with 43 and 37 focal triggers, respectively), the left upper and lower pulmonary veins (PV), and at the right inferior PV (zones 2, 3 and 5). Focal activity in the right atrium was detected in 7 patients during the first mapping and in 5 patients in the second mapping (Supplementary Table 1).

**FIGURE 3:**
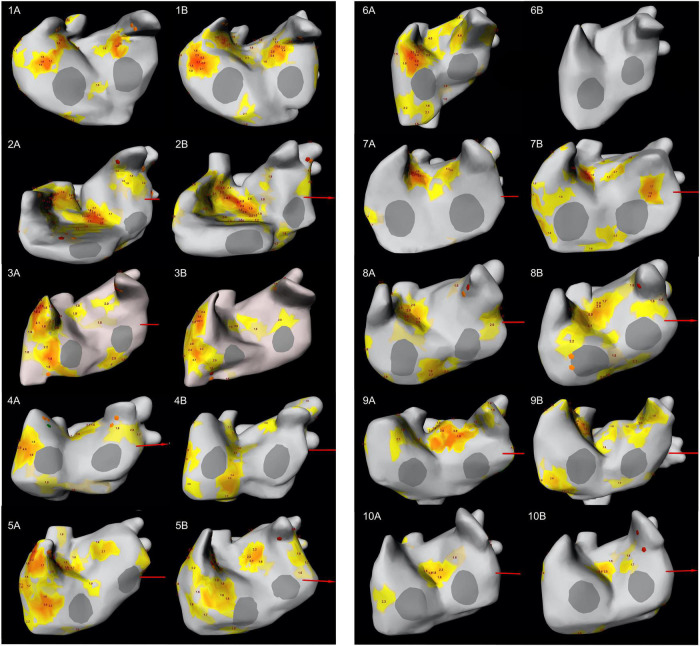
LAO-view showing the distribution of focal and rotor activity of all 10 patients in both mappings. The number indicates the patient number; letter **(A)** indicates the first mapping, letter **(B)** indicates the second mapping. Focal activity can be recognized as orange diamond while rotor activity is depicted as yellow/orange area. The number at the area’s center indicates the number of rotations. LAO, left anterior oblique.

**FIGURE 4:**
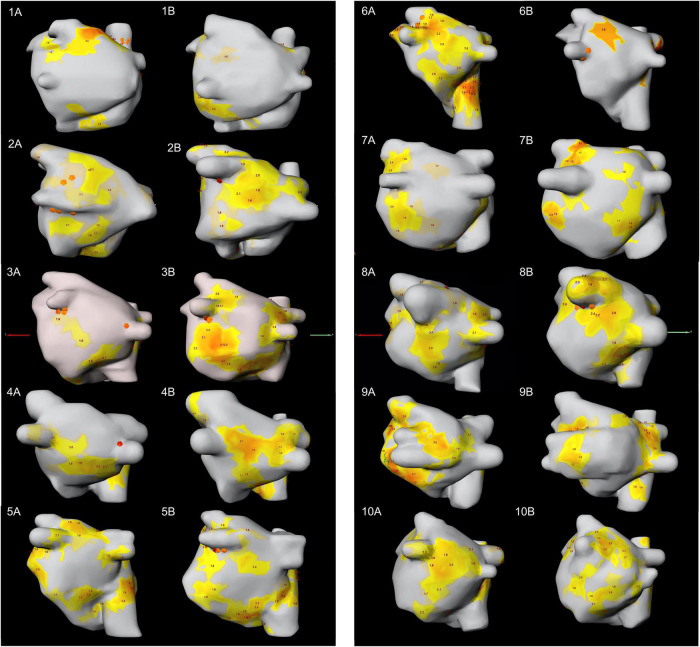
PA-view showing the distribution of focal and rotor activity of all 10 patients in both mappings. The number indicates the patient number; letter **(A)** indicates the first mapping, letter **(B)** indicates the second mapping. PA, posterior anterior.

Rotational driver activity was documented consistently in all patients, in all ECGI mappings and always involved both atria (Figures 3, 4). In zone 5 (right inferior PV), rotor activity was seen in every patient during both mappings (Table 2 and Supplementary Table 1). Zones 9 and 14 (upper half of right atrium and antero-superior part of left atrium, respectively) showed rotor activity in 95% of all examinations. The highest cumulative amount of rotational activity was detected in the upper half of the right atrium (225; zone 9), followed by zones 5, 3 and 10 (inferior right and left PV and lower half of right atrium showed 122, 113 and 112 rotors, respectively).

Interestingly, dynamic change in the location and type of arrhythmogenic activity was observed in all patients when comparing the two consecutive ECGI mappings. Supplementary Tables 2, 3 show the distribution of focal and rotor activity per zone for each patient.

### Quantitative changes

In summary, some numerical differences between the two mappings were detected in most subjects (Supplementary Tables 2, 3). Overall however, quantitative analysis of rotor and focal activities showed no statistically significant difference between the two mappings (median total number of focal activities 8 (IQR: 1–16) versus 6 (IQR: 2–12); *p* = 0.68, median total number of rotor activities 48 (IQR: 44–67) versus 55 (IQR: 44–61); *p* = 0.30).

The median difference of the total number of focal activities at the second mapping subtracting the total number at the first mapping was −0.5 (IQR: −4–3; minimum: −14; maximum: 13).

The median difference of the total number of rotor activities at the second mapping subtracting the total number at the first mapping was 6 (IQR: −4–12; minimum: −64; maximum: 17).

**FIGURE 5:**
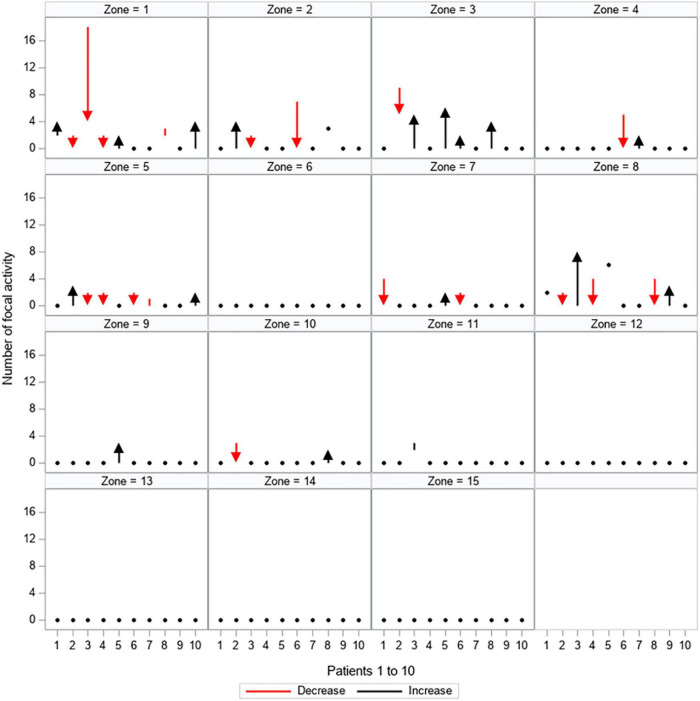
Quantitative changes in focal activity. All 15 zones are depicted separately, showing the load of focal activity (y-axis) for every patient (patients 1–10 are displayed on the x-axis). The dynamic between the first and second mapping is shown: red arrows and lines represent a decrease in the amount of focal activity, black arrows and lines represent an increase in the amount of focal activity, black dots represent no change between the first and second mapping.

**FIGURE 6:**
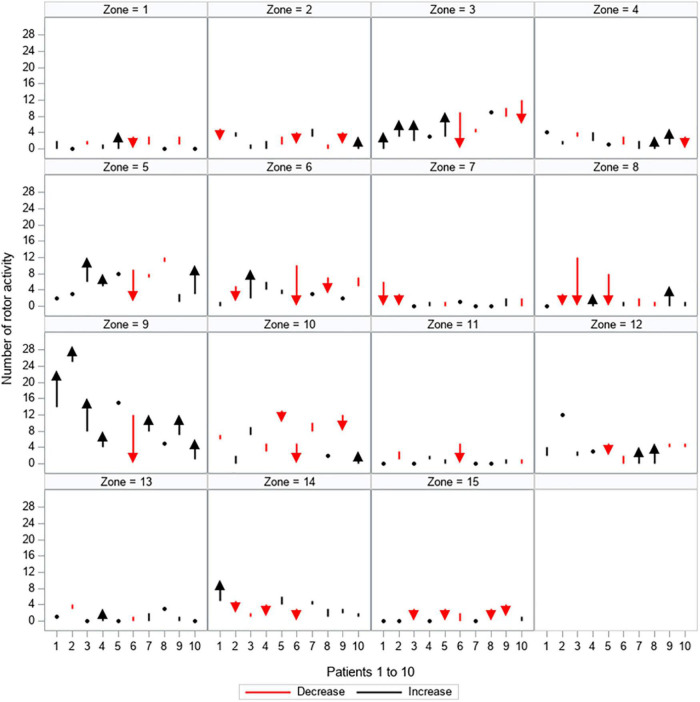
Quantitative changes in rotor activity. All 15 zones are depicted separately, showing the load of rotor activity (y-axis) for every patient (patients 1–10 are displayed on the x-axis). The dynamic between the first and second mapping is shown: red arrows and lines represent a decrease in the amount of rotor activity, black arrows and lines represent an increase in the amount of rotor activity, black dots represent no change between the first and second mapping.

The proportion of change between the first and second mapping is depicted for every patient in Figures 5, 6 and Supplementary Figures 2, 3. Supplementary Table 4 shows the proportion of patients showing quantitative changes in the number of activities per zone.

Table 3 shows which locations (zones) were mostly subject to change (left/entered) by AF drivers over time.

**TABLE 3:** Changes per zone.

	Focal activity		Rotor activity	
		
Zone	No. of patients (%) with changes	Increase (+)/Decrease (−) of focal activity	No. of patients (%) with changes	Increase (+)/Decrease (−) of rotor activity
1 2 3 4 5 6 7 8 9 10 11 12 13 14 15	7 (70) 3 (30) 5 (50) 2 (20) 6 (60) 0 (0) 3 (30) 5 (50) 1 (10) 2 (20) 1 (10) 0 (0) 0 (0) 0 (0) 0 (0)	+ 8/−19 +4/−9 +17/−4 +2/−5 +5/−7 +0/−0 +2/−6 +11/−10 +3/−0 +2/−3 +1/−0 +0/−0 +0/−0 +0/−0 +0/−0	7 (70) 10 (100) 8 (80) 8 (80) 7 (70) 8 (80) 6 (60) 9 (90) 8 (80) 9 (90) 6 (60) 8 (80) 5 (50) 10 (100) 6 (60)	+ 7/−9 +9/−12 +19/−18 +12/−6 +18/−10 +11/−20 +3/−12 +10/−26 +39/−12 +7/−18 +3/−8 +12/−7 +6/−2 +12/−10 +1/−14

Values are *n* (%) unless indicated otherwise.

Seventy percent of the patients showed a change of focal activity in zone 1 (left atrial appendage) and 60% in zone 5 (region of the right lower PV).

Rotor activity was subject to more changes and mostly affected the following zones: Zone 2 and 14 (the left upper PV region and the upper anterior region of the left atrium showed changes in all patients (100%). Changes were detected in 90% of the patients in zones 8 and 10 (right atrial appendage and the lower region of the right atrium).

Interestingly, the absolute number of driver variability differed in different zones, most quantitative changes of focal activity was found in zone 1 (left atrial appendage), zone 3 (region of the left lower PV) and zone 8 (right atrial appendage).

While most quantitative changes of rotor activity were found at zone 3 (region of the left lower PV) and the upper half of the right atrium and the atrial appendage (zones 9 and 8). Details for each zone are listed in Table 3.

### Anatomical changes

Importantly, while the total number of focal and rotor activities may be stable when comparing the two mappings, their anatomical region and precise location differed; in other words: dynamic changes in the arrhythmogenic regions were observed.

To determine the number of patients with changes between the first and the second mapping, measurements were categorized as “focal activity present” vs. “no focal activity present,” and “rotor activity present” vs. “no rotor activity present,” at each zone for each patient. Patients with “activity present” in the first mapping and “no activity present” in the second mapping, or vice versa, in at least one zone were considered patients with a change.

In all patients, changes were observed with regards to the presence of focal activity [100% showed changes, 95%CI (69.2%–100%), *p* < 0.001], rotor activity [100% showed changes, 95%CI (69.2%–100%), *p* < 0.001], and therefore, both focal and rotor activity [100% showed changes, 95%CI (69.2%–100%), *p* < 0.001].

The distribution of rotor and focal activity for each patient and map is depicted in Figures 3, 4. We observed certain consistent “hot-spots” in several patients (e.g., seen in Figure 3: zone 12, the anterior interatrial groove or septal region), however, all patients clearly showed anatomical changes when comparing the mappings.

## Discussion

Long-standing persistent atrial fibrillation is considered the most complex form of AF with a significant degree of atrial tissue remodeling (18). In today’s practice, the majority of patients with symptomatic LSpAF undergo catheter-based mapping that often serves as the basis for a catheter-based intervention. This approach requires significant sedation to allow catheter maneuvers and a transeptal approach. As a result, catheter-ablation is performed at the same time as the mapping. The potential limitations of this approach are that the sedation required alters and suppresses arrhythmia and their invasiveness limits the admissible number of tests to capture the mechanism of AF more accurately. Moreover, catheter-based mapping techniques are almost always followed by an invasive ablative procedure which can result in scarring that may, in turn, alter a later mapping. The challenge of sedation and invasiveness is avoided by body-surface mapping with ECGI, which only requires wrapping the chest in a multi-electrode vest and can therefore be performed as often as needed. This mapping technique also provides a simultaneous view of the electrical activity in both atria on a beat-to-beat basis, albeit with less spatial resolution than catheter-mapping (13).

One of the reasons catheter ablation for LSpAF can fail is the inability to create complete and permanent lesions of conduction block in the desired areas of the atrium (19). Contiguous, uniformly transmural atrial lesions are difficult to create with the tip of a long catheter in a beating, working heart (20). Another potential source of failure for LSpAF treatment with catheter ablation is that following initial pulmonary vein isolation, the localization and ablation of alleged focal AF drivers (21) outside the pulmonary veins are based solely on intraprocedural mappings. Such an approach assumes that the location of these allegedly focal AF drivers is fixed, i.e., does not change with time. The present study was designed to confirm or refute the validity of this assumption. ECGI would facilitate a better understanding of AF driver stability over time and provide a potential explanation for the special challenges in ablating patients with LSpAF.

At this point we want to stress that our study was not primarily designed to support or oppose any theory of sustaining mechanisms underlying AF, like the *multiple wavelet* (22) or *localized driver* (23) theory. Recent studies have shown that the electrophysiological mechanism responsible for AF is far more complex than had been assumed (24, 25). For instance, Alessie’s group has shown that the atrial wall can function as a 3D-structure; epi- and endocardial conduction dissociations, resulting in asynchronous activation, entail a highly complex mechanism (26). Inherent to LSpAF is substantial structural remodeling of the atrial tissue, which probably facilitates the formation and abundance of reentrant circuits (27).

The present study is unique as we carried out two consecutive ECGI mappings in each patient, allowing a minimum interval of 7 days between both mappings. The patients analyzed by us were exclusively patients with LSpAF to guarantee consistency. No therapy changes were allowed during or between the two mappings in order to avoid therapy-induced alterations.

In our study, topographical changes in both focal and rotor activities were found in all patients. We demonstrated that several patients showed certain permanent “hot-spots” of localized driver regions, e.g., in the septal area, but independently of these, every patient clearly showed changes in driver location during the second examination, resulting in a different location of drivers and different patterns of activity during AF in the same patients. At the same time, the overall quantitative number of drivers remained stable between the first and second mapping. However, individual zones showed a high amount of quantitative changes (increase/decrease) of driver activity. Most changes of focal activity were found in the left atrial appendage, the region of the left lower pulmonary vein and the right atrial appendage. Most changes in rotor activity were found also at the left lower PV region, the upper half of the right atrium and the right atrial appendage. This leads us to believe that the insight gained by every mapping in LSpAF can therefore only be considered as a snapshot view of a complex and dynamic electrophysiological state. This may provide an explanation of the frequent failure of procedures that target allegedly localized drivers which are based on intra-procedural mapping showing the characteristics of AF only on that particular day.

The other important finding of our study is that bi-atrial electrophysiological pathology was observed in all subjects, in all ECGI mappings. This study’s consistent findings of bi-atrial activity in AF did not come as a surprise to us, as previous studies in a similar group of patients with non-paroxysmal AF already demonstrated such pathophysiology for AF (28).

Our results explain an important aspect for the success of anatomically-based bi-atrial approaches for LSpAF such as the Cox-maze procedure that, according to some reports, gains the highest single-procedure success rate (29–31). An anatomical approach, unlike the map-and-ablate approach, may have the advantage of addressing the atrial substrate in a more global way. Moreover, the fact that in our patient collective bi-atrial electrophysiological activity was observed in all mappings serves to explain the lower success rate of ablative approaches that are limited to the left atrium only in patients with LSpAF (30, 32). It should be pointed out, that experimental and clinical findings proved clearly, that all MAZE lesion sets need to be performed in an effort to treat persistent or long-standing persistent AF successfully. It is essential for us to realize that in many cases, and especially when a unidirectional unipolar RF is being used, the lines will be found to be inconsistent and often incomplete. Therefore, the recurrence rate may be equally high whether surgeons are performing a full biatrial lesion set or just PVI using such technologies. Our results may suggest that further considerations are required before addressing only the left atrium in such patients, and good planning together with a cardiac electrophysiologist is crucial in order to improve outcomes. The present findings are unique and deserve further assessment; obviously, a larger multicenter study is needed to better represent the surgical patient population and may help to choose the best surgical ablation approach.

However, irrespective of whether or not the sites of localized rotor activity are the actual drivers that sustain PEAF and LSpAF, the present study suggests that multiple mapping prior to an intervention may augment our understanding of the pathophysiology of AF in a given patient. It may therefore help to identify the zones with permanent pathologies versus those that are subject to change. This will in turn lead to modification of the ablation strategy and may improve the results in such a challenging group of patients. Following the conclusion of this study, it may be worth starting a renewed discussion about the role of anatomical ablation approaches, such as the bi-atrial Cox-maze procedure, not only in surgery but potentially also in catheter ablation.

### Study limitations

This electrophysiological study was performed in a relatively small cohort with concomitant LSpAF, although, for this kind of study, the number of patients is acceptable. Our results may not be directly applicable to paroxysmal AF. There are known limitations to non-invasive mapping. ECGI determines the heart’s electrical sources for a given body-surface potential distribution. This is also known as the inverse problem, which is referred to as ill posed. There is no unique solution and the solution is very sensitive to little changes in the input, due to measurement noise and inaccuracies in the estimation of the cardiac-torso geometry (13). This is one of the main reasons why ECGI has been criticized for being inaccurate in its ability to precisely identify drivers; however, Cuculich et al. have proven an accuracy of 6 mm (33). The main limitation of our study was that we did not additionally use invasive measurement techniques to acquire also local intracardiac electrograms. However, our aim was not to perform a precise measurement of the allegedly fixed location of drivers, but rather to show that their location varies; and for this, our method was entirely adequate.

Nevertheless, mapping was successfully performed in all patients without technical difficulties, yielding excellent quality data. Our results proved to be highly consistent—all patients showed changes in the distribution of AF drivers. If our findings are validated by other mapping modalities, specifically endocardial mapping, repeated invasive mapping procedures would be necessary. We believe that possible disadvantages of our technique, such as the somewhat lower subsurface resolution compared to the resolution gained with invasive mapping, are offset by the possibility of repeated mapping without the need for sedation, which is especially important in vulnerable patients.

## Conclusion

This clinical electrophysiological study demonstrates bi-atrial pathophysiology and changes in the localization of rotor and focal activity in patients with LSpAF who had two consecutive mappings. No quantitative differences were identified. These results corroborate the importance of an anatomical approach to ablation when treating LSpAF.

## Data availability statement

The original contributions presented in this study are included in the article/Supplementary material, further inquiries can be directed to the corresponding author/s.

## Ethics statement

The studies involving human participants were reviewed and approved by Ethics Review Board of the Medical University of Vienna. The patients/participants provided their written informed consent to participate in this study.

## Author contributions

EO-J was responsible for conceptualization and design, analysis and interpretation, data collection, statistical analysis, writing, critical revision of the manuscript, and gave final approval. JC, ME, and NA were responsible for conceptualization and design, analysis and interpretation, writing, supervision, critical revision of the manuscript, and gave final approval. SK and PW were responsible for data collection, critical revision of the manuscript, and gave final approval. AK was responsible for conceptualization and design, analysis and interpretation, statistical analysis, writing, critical revision of the manuscript, and gave final approval. PA and MM were responsible for analysis and interpretation, critical revision of the manuscript, and gave final approval. AS was responsible for data collection, provided resources, technical and logistic support, critical revision of the manuscript, and gave final approval. GL was responsible for providing resources, supervision, critical revision of the manuscript, and gave final approval. All authors contributed to manuscript revision, read, and approved the submitted version.
