# Supplementary material for: Dynamic electrophysiological mechanism in patients with long-standing persistent atrial fibrillation
Source: Front Cardiovasc Med. 2022 Sep 29;9:953622. doi: 10.3389/fcvm.2022.953622 (PMC9556291; doi:10.3389/fcvm.2022.953622)
Supplement: Supplementary file 1 [file Data_Sheet_1.docx]

Supplementary Material

**Table of Contents**

**Figures**

Supplementary Figure 1: Line diagrams of quantitative changes in focal activity. 2

Supplementary Figure 2: Line diagrams of quantitative changes in rotor activity. 3

**Tables**

Supplementary Table 1: Patients and zones. 4

Supplementary Table 2: Focal activity at first and second mapping. 6

Supplementary Table 3: Rotor activity at first and second mapping. 7

Supplementary Table 4: Percentage of patients showing changes. 8


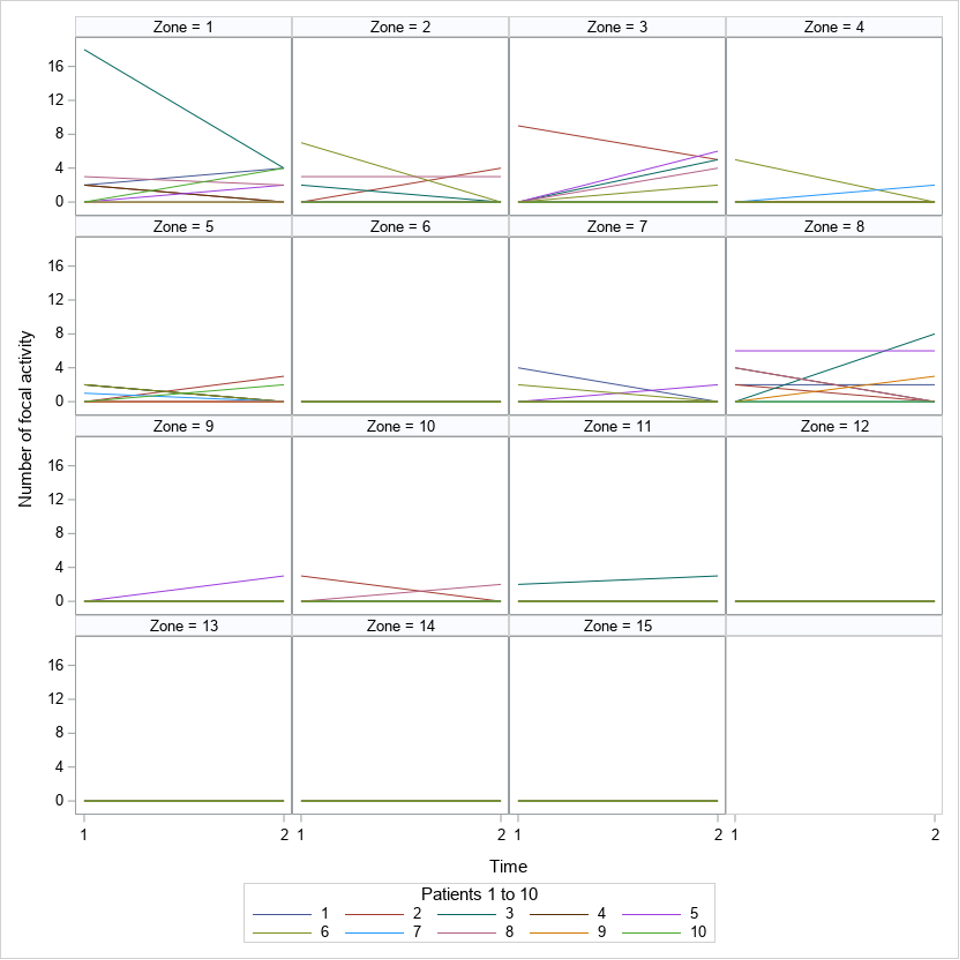


Supplementary Figure 1: Line diagrams of quantitative changes in focal activity. All 15 zones are depicted separately, showing the load of focal activity (y axis) for every patient (patients 1 to 10 are displayed in different colors, see legend). The dynamic between the first and second mapping (x axis) is shown: a declining line represents a decrease in the amount of focal activity, an ascending line represent an increase in the amount of focal activity, a horizontal line represents no change between the first and second mapping.


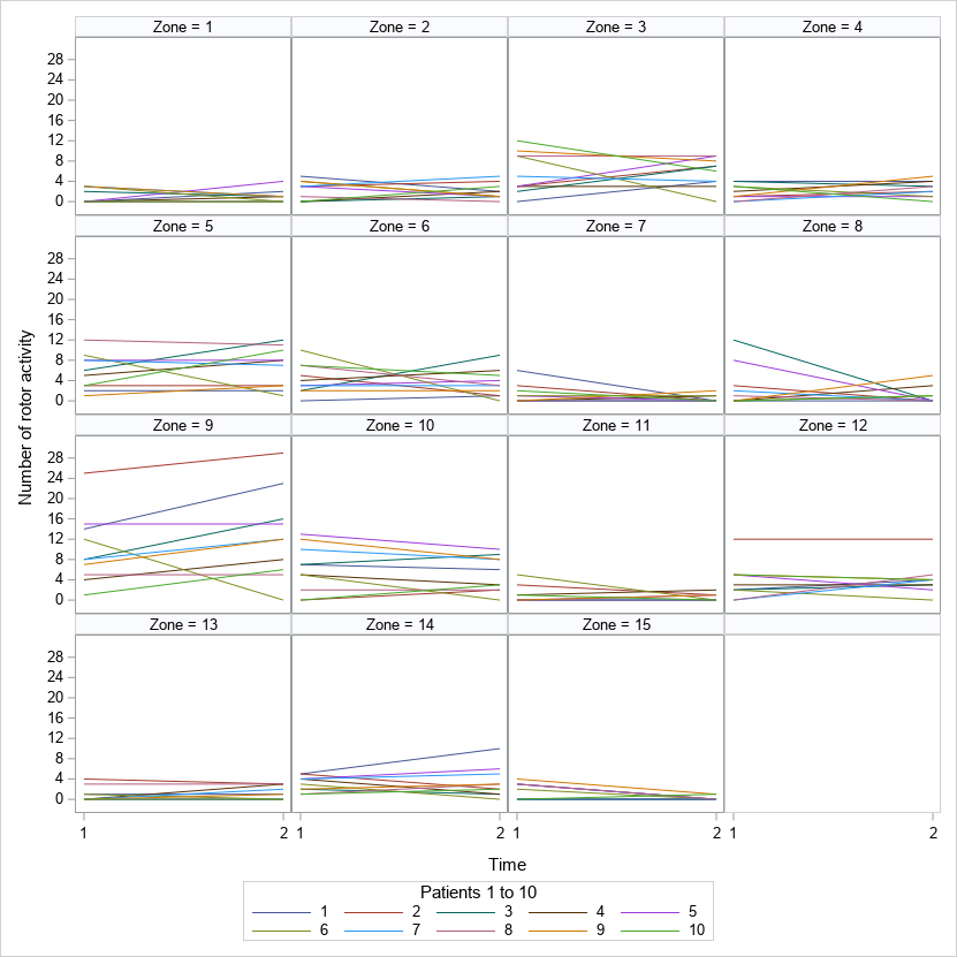


Supplementary Figure 2: Line diagrams of quantitative changes in rotor activity. All 15 zones are depicted separately, showing the load of rotor activity (y axis) for every patient (patients 1 to 10 are displayed in different colors, see legend). The dynamic between the first and second mapping (x axis) is shown: a declining line represents a decrease in the amount of focal activity, an ascending line represent an increase in the amount of focal activity, a horizontal line represents no change between the first and second mapping.

| **Supplementary Table 1: Patients and zones** | | | |
| --- | --- | --- | --- |
|  | **All mappings**  **(*n*= 20)** | **First mapping**  **(*n*= 10)** | **Second**  **mapping**  **(*n*= 10)** |
| **Focal activity** | | | |
| Patients with focal activity | 17 (85) | 8 (80) | 9 (90) |
| **Number of patients with focal activities per zone** | | | |
| Zone 1 | 10 (50) | 5 (50) | 5 (50) |
| Zone 2 | 5 (25) | 3 (30) | 2 (20) |
| Zone 3 | 6 (30) | 1 (10) | 5 (50) |
| Zone 4 | 2 (10) | 1 (10) | 1 (10) |
| Zone 5 | 6 (30) | 4 (40) | 2 (20) |
| Zone 6 | 0 (0) | 0 (0) | 0 (0) |
| Zone 7 | 3 (15) | 2 (20) | 1 (10) |
| Zone 8 | 9 (45) | 5 (50) | 4 (40) |
| Zone 9 | 1 (5) | 0 (0) | 1 (10) |
| Zone 10 | 2 (10) | 1 (10) | 1 (10) |
| Zone 11 | 2 (10) | 1 (10) | 1 (10) |
| Zone 12 | 0 (0) | 0 (0) | 0 (0) |
| Zone 13 | 0 (0) | 0 (0) | 0 (0) |
| Zone 14 | 0 (0) | 0 (0) | 0 (0) |
| Zone 15 | 0 (0) | 0 (0) | 0 (0) |
| **Rotor activity** | | | |
| Patients with rotor activity | 20 (100) | 10 (100) | 10 (100) |
| **Number of patients with rotor activities per zone** | | | |
| Zone 1 | 10 (50) | 3 (30) | 6 (60) |
| Zone 2 | 16 (80) | 7 (70) | 9 (90) |
| Zone 3 | 18 (90) | 9 (90) | 9 (90) |
| Zone 4 | 17 (85) | 8 (80) | 9 (90) |
| Zone 5 | 20 (100) | 10 (100) | 10 (100) |
| Zone 6 | 18 (90) | 9 (90) | 9 (90) |
| Zone 7 | 8 (40) | 5 (50) | 3 (30) |
| Zone 8 | 9 (45) | 5 (50) | 4 (40) |
| Zone 9 | 19 (95) | 10 (100) | 9 (90) |
| Zone 10 | 17 (85) | 8 (80) | 9 (90) |
| Zone 11 | 8 (40) | 4 (40) | 4 (40) |
| Zone 12 | 17 (85) | 8 (80) | 9 (90) |
| Zone 13 | 10 (50) | 4 (40) | 6 (60) |
| Zone 14 | 19 (95) | 10 (100) | 9 (90) |
| Zone 15 | 7 (35) | 5 (50) | 2 (20) |

Values are n (%) unless indicated otherwise.

| **Supplementary Table 2: Focal activity at first and second mapping** | | | | | | | | | | | | | | | | | |
| --- | --- | --- | --- | --- | --- | --- | --- | --- | --- | --- | --- | --- | --- | --- | --- | --- | --- |
| **Patient no.** | **Mapping** | **Z 1** | **Z 2** | **Z 3** | **Z 4** | **Z 5** | **Z 6** | **Z 7** | **Z 8** | **Z 9** | **Z 10** | **Z 11** | **Z 12** | **Z 13** | **Z 14** | **Z 15** | **∑** |
|  |  |  |  |  |  |  |  |  |  |  |  |  |  |  |  |  |  |
| 1 | First | 2 |  |  |  |  |  | 4 | 2 |  |  |  |  |  |  |  | 8 |
| 1 | Second | 4 |  |  |  |  |  |  | 2 |  |  |  |  |  |  |  | 6 |
| 2 | First | 2 |  | 9 |  |  |  |  | 2 |  | 3 |  |  |  |  |  | 16 |
| 2 | Second |  | 4 | 5 |  | 3 |  |  |  |  |  |  |  |  |  |  | 12 |
| 3 | First | 18 | 2 |  |  | 2 |  |  |  |  |  | 2 |  |  |  |  | 24 |
| 3 | Second | 4 |  | 5 |  |  |  |  | 8 |  |  | 3 |  |  |  |  | 20 |
| 4 | First | 2 |  |  |  | 2 |  |  | 4 |  |  |  |  |  |  |  | 8 |
| 4 | Second |  |  |  |  |  |  |  |  |  |  |  |  |  |  |  | 0 |
| 5 | First |  |  |  |  |  |  |  | 6 |  |  |  |  |  |  |  | 6 |
| 5 | Second | 2 |  | 6 |  |  |  | 2 | 6 | 3 |  |  |  |  |  |  | 19 |
| 6 | First |  | 7 |  | 5 | 2 |  | 2 |  |  |  |  |  |  |  |  | 16 |
| 6 | Second |  |  | 2 |  |  |  |  |  |  |  |  |  |  |  |  | 2 |
| 7 | First |  |  |  |  | 1 |  |  |  |  |  |  |  |  |  |  | 1 |
| 7 | Second |  |  |  | 2 |  |  |  |  |  |  |  |  |  |  |  | 2 |
| 8 | First | 3 | 3 |  |  |  |  |  | 4 |  |  |  |  |  |  |  | 10 |
| 8 | Second | 2 | 3 | 4 |  |  |  |  |  |  | 2 |  |  |  |  |  | 11 |
| 9 | First |  |  |  |  |  |  |  |  |  |  |  |  |  |  |  | 0 |
| 9 | Second |  |  |  |  |  |  |  | 3 |  |  |  |  |  |  |  | 3 |
| 10 | First |  |  |  |  |  |  |  |  |  |  |  |  |  |  |  | 0 |
| 10 | Second | 4 |  |  |  | 2 |  |  |  |  |  |  |  |  |  |  | 6 |
| **∑** |  | 43 | 19 | 31 | 7 | 12 | 0 | 8 | 37 | 3 | 5 | 5 | 0 | 0 | 0 | 0 | **170** |

Values are *n.* ∑: Sum; Z: Zone.

| **Supplementary Table 3: Rotor activity at first and second mapping** | | | | | | | | | | | | | | | | | |
| --- | --- | --- | --- | --- | --- | --- | --- | --- | --- | --- | --- | --- | --- | --- | --- | --- | --- |
| **Patient no.** | **Mapping** | **Z 1** | **Z 2** | **Z 3** | **Z 4** | **Z 5** | **Z 6** | **Z 7** | **Z 8** | **Z 9** | **Z 10** | **Z 11** | **Z 12** | **Z 13** | **Z 14** | **Z 15** | **∑** |
|  |  |  |  |  |  |  |  |  |  |  |  |  |  |  |  |  |  |
| 1 | First |  | 5 |  | 4 | 2 |  | 6 |  | 14 | 7 |  | 2 | 1 | 5 |  | 46 |
| 1 | Second | 2 | 2 | 4 | 4 | 2 | 1 |  |  | 23 | 6 |  | 4 | 1 | 10 |  | 59 |
| 2 | First |  | 3 | 3 | 1 | 3 | 5 | 3 | 3 | 25 |  | 3 | 12 | 4 | 5 |  | 70 |
| 2 | Second |  | 4 | 7 | 2 | 3 | 1 |  |  | 29 | 2 | 1 | 12 | 3 | 2 |  | 66 |
| 3 | First | 2 |  | 2 | 4 | 6 | 2 |  | 12 | 8 | 7 |  | 2 |  | 2 | 3 | 50 |
| 3 | Second | 1 | 1 | 7 | 3 | 12 | 9 |  |  | 16 | 9 |  | 3 |  | 1 |  | 62 |
| 4 | First |  |  | 3 | 2 | 5 | 4 |  |  | 4 | 5 | 1 | 3 |  | 4 |  | 31 |
| 4 | Second | 1 | 2 | 3 | 4 | 8 | 6 | 1 | 3 | 8 | 3 | 2 | 3 | 3 | 1 |  | 48 |
| 5 | First |  | 3 | 3 | 1 | 8 | 3 | 1 | 8 | 15 | 13 |  | 5 |  | 4 | 3 | 67 |
| 5 | Second | 4 | 1 | 9 | 1 | 8 | 4 |  |  | 15 | 10 | 1 | 2 |  | 6 |  | 61 |
| 6 | First | 3 | 4 | 9 | 3 | 9 | 10 | 1 |  | 12 | 5 | 5 | 2 | 1 | 3 | 2 | 69 |
| 6 | Second |  | 1 |  | 1 | 1 |  | 1 | 1 |  |  |  |  |  |  |  | 5 |
| 7 | First | 3 | 3 | 5 |  | 8 | 3 |  | 2 | 8 | 10 |  |  |  | 4 |  | 46 |
| 7 | Second | 1 | 5 | 4 | 2 | 7 | 3 |  |  | 12 | 8 |  | 4 | 2 | 5 |  | 53 |
| 8 | First |  | 1 | 9 |  | 12 | 7 |  | 1 | 5 | 2 |  |  | 3 | 1 | 3 | 44 |
| 8 | Second |  |  | 9 | 3 | 11 | 3 |  |  | 5 | 2 |  | 5 | 3 | 3 |  | 44 |
| 9 | First | 3 | 4 | 10 | 1 | 1 | 2 |  |  | 7 | 12 |  | 5 |  | 2 | 4 | 51 |
| 9 | Second | 1 | 1 | 8 | 5 | 3 | 2 | 2 | 5 | 12 | 8 | 1 | 4 | 1 | 3 | 1 | 57 |
| 10 | First |  |  | 12 | 3 | 3 | 7 | 2 |  | 1 |  | 1 | 5 |  | 1 |  | 35 |
| 10 | Second |  | 3 | 6 |  | 10 | 5 |  | 1 | 6 | 3 |  | 4 |  | 2 | 1 | 41 |
| **∑** |  | 21 | 43 | 113 | 44 | 122 | 77 | 17 | 36 | 225 | 112 | 15 | 77 | 22 | 64 | 17 | **1005** |

Values are *n.* ∑: Sum; Z: Zone.

| **Supplementary Table 4: Percentage of patients showing quantitative changes** | | | | | | |
| --- | --- | --- | --- | --- | --- | --- |
|  | **Focal activity** | | | **Rotor activity** | | |
| Zone | increased | no change | decreased | increased | no change | decreased |
| 1 2 3 4 5 6 7 8 9 10 11 12 13 14 15 | 30%  10% 40% 10% 20%  0% 10% 20%  10% 10% 10% 0% 0% 0% 0% | 30%  70% 50% 80% 40% 100% 70% 50% 90% 80% 90% 100% 100% 100% 100% | 40%  20% 10% 10% 40%  0% 20% 30%  0%  10%  0%  0% 0% 0% 0% | 30% 50%  40% 50% 40% 40% 20%  40%  70% 30%  30%  40% 30% 60% 10% | 30%  0% 20% 20% 30% 20% 40% 10% 20% 10% 40% 20% 50% 0% 40% | 40%  50% 40% 30% 30%  40% 40%  50% 10% 60% 30%  40% 20% 40% 50% |

Percentages represent the proportion of patients showing changes in the number of drivers per
zone.
